# Supplementary figures and images for: Mycorrhizal fungi reduce the photosystem damage caused by drought stress on Paris polyphylla var. yunnanensis
Source: PLoS One. 2024 Apr 18;19(4):e0294394. doi: 10.1371/journal.pone.0294394 (PMC11025924; doi:10.1371/journal.pone.0294394)

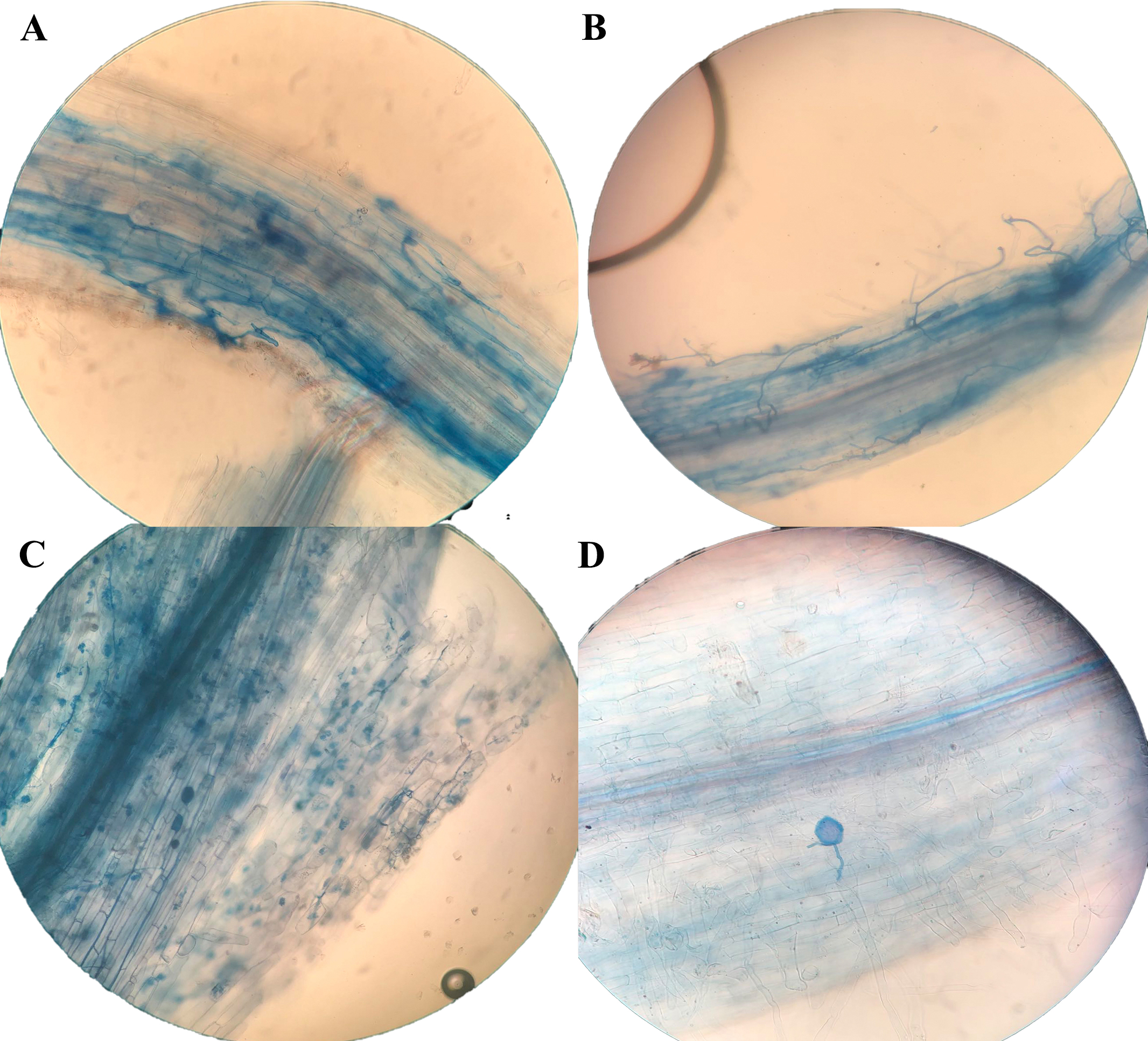

Supplement: S1 Fig — (A structure of mycorrhizal mycorrhizal infestation by Ge under normal moisture, B structure of mycorrhizal infestation by Po under normal moisture, C structure of mycorrhizal infestation by Ge under drought stress, D structure of mycorrhizal fungal inoculation without tufts under normal moisture). (JPG) [file pone.0294394.s001.jpg]
