# Supplementary material for: Mycorrhizal fungi reduce the photosystem damage caused by drought stress on Paris polyphylla var. yunnanensis
Source: PLoS One. 2024 Apr 18;19(4):e0294394. doi: 10.1371/journal.pone.0294394 (PMC11025924; doi:10.1371/journal.pone.0294394)
Supplement: Attachment — Submitted filename: Response to Reviewers.docx [file pone.0294394.s002.docx]

Dear Jeunarine Repe Flores

We have carefully reviewed your letter and have corrected the manuscript accordingly to your request.

Firstly, the article formatting and typos were corrected according to PLOS ONE's manuscript guidelines.

The contribution of the project sponsor was described in detail.

Reply to Reviewer #1

The research background introduction of the article was added, relevant references [17,18,27,28] were added, and more information on the relationship between drought and photosynthesis was provided.

The sentence order of the last paragraph of the introduction was changed, and p.py was placed in the first sentence. The importance of this study is highlighted.

Additions were made to address the relevant measurements of PSI and PSII in the material methods, and typos and incorrect formatting were revised.

The incorrect presentation of 4=lts was modified.

The usage of OJIP curves was unified.

Changed the treatment numbers in Table 1, WX, WYL, DX, and DYL to WGe, WPo, DGe, and DPo, respectively.

Corrected the table serial numbers, the previous manuscript incorrectly labeled Table 2 as Table 3.

Reply to Reviewer #2

Added explanation of some technical terms after the abstract.

Harmonized the expressions of AMF plants and AM plants, and changed AM plants to AMF plants uniformly.

Revised the misrepresentation of D.P.py.

Drought conditions are mainly adjusted by moisture meter, according to the measurement results, the soil water content under drought and sufficient moisture conditions is controlled within the corresponding range. For example, drought soils are measured at 15-18% moisture by the moisture meter, and 33-35% for adequate moisture treatment soils.

Images of root colonization of P.py are provided in the additional images.

These are the main revisions of this paper, and thank you again for your reply.

Kind regards,

Can Huang
