# Supplementary material for: Mycorrhizal fungi reduce the photosystem damage caused by drought stress on Paris polyphylla var. yunnanensis
Source: PLoS One. 2024 Apr 18;19(4):e0294394. doi: 10.1371/journal.pone.0294394 (PMC11025924; doi:10.1371/journal.pone.0294394)
Supplement: Attachment — Submitted filename: Review.docx [file pone.0294394.s003.docx]

Using *Paris polyphylla* and arbuscular mycorrhiza fungi (AMF), the authors examined AMF colonization rate, relative water content, chlorophyll content and photosynthesis under drought stress. The experiments were designed well and executed efficiently. The methods and results are well justified towards the primary goal of this study. This study could be interesting to the Plant cell, Tissue and Organ Culture audience. A couple of minor things need to be solved before it is published.

1. In last sentence it is written that it is necessary to study the relationship between Dianthus, AMF and photosynthesis. The term Dianthus is not used anywhere. Is it Dianthus or P.Py.?
2. In materials and methods, how P.py seedlings were planted without 1 Kg of soil mixture. Sentence error?
